# Editorial: Proteoglycans and Glycosaminoglycan Modification in Immune Regulation and Inflammation

**DOI:** 10.3389/fimmu.2020.595867

**Published:** 2020-10-15

**Authors:** Rogier M. Reijmers, Linda Troeberg, Megan S. Lord, Aaron C. Petrey

**Affiliations:** ^1^LUMICKS B.V., Amsterdam, Netherlands; ^2^Norwich Medical School, University of East Anglia, Norwich, United Kingdom; ^3^Graduate School of Biomedical Engineering, University of New South Wales Sydney, Sydney, NSW, Australia; ^4^University of Utah Molecular Medicine Program, Salt Lake City, UT, United States; ^5^Division of Microbiology and Immunology, Department of Pathology, University of Utah, Salt Lake City, UT, United States

**Keywords:** glycosaminoglycan (GAG), proteoglycans (PG), immune response, inflammation, heparan sulfate (HS), chondroitin sulfate (CS), dermatan sulfate (DS), hyaluronan

While many publications cover proteoglycan and glycosaminoglycan (GAG) research each year, to date there is still limited knowledge about the specific roles of these macromolecules in the regulation of immune responses and homeostasis. More specifically, the transcriptional control is understudied and largely unknown (1). For this reason, we initiated this Research Topic for Frontiers in Immunology, and challenged scientists to provide findings that uncover novel functions or summarize recent progress that connect these two fields of research. Our collection of 16 manuscripts consists of 7 Original Research papers, one Perspective, one Mini Review, and 7 Reviews covering different aspects of proteoglycan biology in immune processes, which are summarized below.

Proteoglycans are ubiquitously expressed intracellularly, on the cell surface and in the extracellular matrix. Attached to their core are one or a multitude of covalently linked linear anionic GAGs, which are subject to an array of synthetic and post-synthetic enzymatic modifications, leading to an almost infinite combination of configurations, each potentially harboring specific functions (2). As such, precise GAG modifications have been attributed to drive interactions with immune cells, pathogens, and specific protein ligands, while functions have also been attributed to the core proteins. Likewise, the immune system is extremely diverse with many different cell types and immunomodulators, such as chemokines and cytokines, each having specific and unique functions. All of these components need to be kept in strict balance and require tight spatiotemporal regulation to avoid development of autoimmunity or cancer, while combatting a limitless number of pathogens. Understanding this complexity is the greatest challenge in the field and requires detailed structural analysis of GAGs and proteoglycans (3).

Rajarathnam and Desai reviewed recent progress in understanding the molecular basis of chemokine interactions with GAGs and their CXCR1 and CXCR2 receptors. They argue that the range of GAG-binding affinities and geometries confers remarkable selectivity on the *in vivo* phenotype of individual chemokines, and that understanding these interactions is critical for clinical application. Crijns et al. more broadly address chemokine-GAG interactions, and suggest that limiting inflammation using different approaches like chemokine-derived GAG-binding peptides or dominant-negative chemokine mutants, might be beneficial, especially if required to reduce the inflammatory response, rather than completely eliminating it.

Specific effects of GAGs on inflammatory processes were addressed by several authors. For example, Talsma et al. extended our understanding of GAG effects on complement activation by demonstrating that heparin/heparan sulfate oligosaccharides inhibit the lectin pathway via inhibiting the activity of serine protease MASP-2 that cleaves C4. This work may enable the development of glycan-based inhibitors of the lectin pathway with therapeutic value. Arokiasamy, King et al. profiled the glycocalyx of lymphatic vessel endothelial cells, which is less well-understood than the glycocalyx associated with vascular endothelial cells. They showed that the lymphatic glycocalyx of murine cremaster muscles is remodeled in response to TNF-mediated inflammation. Blocking heparanase-mediated degradation of HS had no effect on neutrophil migration through the lymphatic vessels; but reduced lymphatic drainage of interstitial fluid. The responses and function of the lymphatic glycocalyx is thus distinct from that of blood vessels. El Masri et al. explored the role of SULFs in inflammation. Despite clear evidence for critical functions of these enzymes in various pathologies including cancer, the roles of SULFs in inflammatory processes are under-appreciated. To advance this field, further investigations are needed to fully understand their spatial and temporal expression and activity. How this could be strategically advanced in the near future is discussed in this Perspective article.

Use of disease models is progressing our understanding of the role of GAGs in complex *in vivo* scenarios. Pessentheiner et al. summarize the broad role of proteoglycans in obesity-related inflammation. Each class of proteoglycan is discussed, including their enzymatic modifications, and key clinical studies are highlighted implicating proteoglycans as therapeutic targets and biomarkers. Despite limited knowledge of syndecans in obesity-related inflammation, it is clear that syndecans are major players in inflammation. Gopal concisely reviewed the characteristics of syndecans, especially syndecan-1, -2 and -4, explaining their diverse role at the cell surface or as soluble proteins. Arokiasamy, Balderstone et al. focused on the role of syndecan-3 in inflammation and angiogenesis, discussing its impact in several distinct disease types and emphasizing that interactions with the GAG chains make syndecan-3 an inflammatory mediator.

In addition to research on HS, we are beginning to appreciate the roles of chondroitin sulfate (CS) in regulating inflammation. Hatano and Watanabe reviewed the roles of CS produced by antigen presenting cells, in contrast to the more widely studied roles of heparan sulfate (HS) and hyaluronan (HA). Importantly, this review suggests a structure-function relationship for CS based on sulfation with highly sulfated CS, such as CS-C, CS-D, and CS-E, possessing anti-inflammatory properties while the lesser sulfated CS-A possesses both pro- and anti-inflammatory properties. In addition, chain length is a determinant of function with oligosaccharides exhibiting pro-inflammatory properties. Wight et al. provide a comprehensive review on the regulatory roles of the HA-binding proteoglycan versican in the context of immunity and inflammation. The authors summarize how versican and its five different isoforms vary in function, and though not all contain CS chains, each mediates complex roles from development to inflammation by virtue of interacting with a host of diverse immune receptors. They provide insight into cell-type specific functions of versican and highlight how CS containing glycoforms allow versican to function as either a pro- or anti-inflammatory proteoglycan.

Although HA lacks the sulfation shared by the other GAGs, investigation into its regulatory role in inflammatory processes continues to reveal unexpected findings. Dong et al. report a previously unknown link between the HA receptor CD44 and clearance of lung surfactant by alveolar macrophages (AM), which constitutively bind to HA. Loss of CD44 in mice leads to reduced numbers of AMs, and dysregulation of genes involved in cholesterol metabolism. In addition, CD44 deficiency results in a buildup of lipid surfactant, foam-cell AMs, and oxidized lipids which exacerbate lung damage and inflammation. Reeves et al. demonstrate that RSV infection of pediatric human lung fibroblasts results in the formation of an HA-enriched ECM capable of recruiting mast cells and augmenting expression of mast cell proteases. Further, by disrupting HA synthesis or HA binding, the authors demonstrate that increased protease expression depends on mast cell interaction with the HA-matrix produced by infected cells.

Besides the impact on cells and immune modulators, effects of GAGs on bacterial pathogenesis were also explored. Martín et al. report that adhesion of *L. salivarius* Lv72 to HeLa cells induced expression of proteoglycan core proteins but reduced glycosaminoglycan chain biosynthesis by the HeLa cells. In addition, *L. salivarius* Lv72 increased expression of o*ppA* which aids in adhesion to mucosal cells. These results suggest communication between mucosa and microbiota. Galeev et al. demonstrated that proteoglycans not only play a role in bacterial adhesion and uptake by epithelial cells, but that they also play a critical role in the survival of bacteria once internalized via control of intracellular trafficking and endo-lysosomal fusion.

Clinical translation of GAG-based therapies is an exciting challenge. PG545, a synthetic heparanase inhibitor, has been demonstrated to affect tumor viability by preventing angiogenesis and non-neoplastic inflammatory disorders. PG545 acts through competing for GAG binding, and Koliesnik et al. show that it selectively induces Treg development and inhibits the formation of Th17 cells *in vitro* and during *in vivo* delayed type hypersensitivity (DTH). Unexpectedly, these effects were independent of heparanase, but the results support clinical application of PG545 in inflammatory responses associated with DTH. Furthermore, use of human milk glycans such as HA have captured attention for their potential as natural products to enhance immune responses and their safety as therapeutic modalities. Kim and de la Motte summarize several pre-clinical studies highlighting the known roles of HA in intestinal host defense, detailing the receptors which mediate downstream mechanisms activated by exogenous HA including barrier function and anti-microbial effects.

Our Research Topic underscores the diverse role that proteoglycans have in inflammation and how they control different aspects of immunological processes. However, it also demonstrates that there is still much to discover about the specific mechanisms of their action, including transcriptional and translational regulation of proteoglycan and GAG biosynthesis (1).

## Author Contributions

All authors listed have made a substantial, direct and intellectual contribution to the work, and approved it for publication.

## Conflict of Interest

RR was employed by the company LUMICKS. ML is a Director of Glycos Pty Ltd, which is focused on the generation of bioengineered glycosaminoglycans as therapeutics. The remaining authors declare that the research was conducted in the absence of any commercial or financial relationships that could be construed as a potential conflict of interest.

## References

[1] Weiss RJ Spahn PN Toledo AG Chiang AWT Kellman BP Li J. ZNF263 is a transcriptional regulator of heparin and heparan sulfate biosynthesis. Proc Natl Acad Sci USA. (2020) 117:9311–7. 10.1073/pnas.1920880117 32277030 PMC7196839

[2] Bishop JR Schiksz M Esko JD. Heparan sulphate proteoglycans fine-tune mammalian physiology. Nature. (2007) 446:1030–7. 10.1038/nature05817 17460664

[3] Kjellén L Lindahl U. Specificity of glycosaminoglycan-protein interactions. Curr Opin Struct Biol. (2018) 50:101–8. 10.1016/j.sbi.2017.12.011 29455055

